# Near-field photocurrent nanoscopy on bare and encapsulated graphene

**DOI:** 10.1038/ncomms10783

**Published:** 2016-02-26

**Authors:** Achim Woessner, Pablo Alonso-González, Mark B. Lundeberg, Yuanda Gao, Jose E. Barrios-Vargas, Gabriele Navickaite, Qiong Ma, Davide Janner, Kenji Watanabe, Aron W. Cummings, Takashi Taniguchi, Valerio Pruneri, Stephan Roche, Pablo Jarillo-Herrero, James Hone, Rainer Hillenbrand, Frank H. L. Koppens

**Affiliations:** 1 ICFO—Institut de Ciencies Fotoniques, The Barcelona Institute of Science and Technology, 08860 Barcelona, Spain; 2 CIC nanoGUNE, 20018 Donostia-San Sebastian, Spain; 3 Institute of Physics, Chinese Academy of Science, Beijing 100190, China; 4 Department of Mechanical Engineering, Columbia University, New York, New York 10027, USA; 5 Catalan Institute of Nanoscience and Nanotechnology (ICN2), CSIC and The Barcelona Institute of Science and Technology, Campus UAB, 08193 Barcelona, Spain; 6 Department of Physics, Massachusetts Institute of Technology, Cambridge, Massachusetts 02139, USA; 7 National Institute for Materials Science, 1-1 Namiki, Tsukuba 305-0044, Japan; 8 ICREA-Institució Catalana de Recerca i Estudis Avançats, 08010 Barcelona, Spain; 9 CIC nanoGUNE and UPV/EHU, 20018 Donostia-San Sebastian, Spain; 10 IKERBASQUE, Basque Foundation for Science, 48011 Bilbao, Spain

## Abstract

Optoelectronic devices utilizing graphene have demonstrated unique capabilities and performances beyond state-of-the-art technologies. However, requirements in terms of device quality and uniformity are demanding. A major roadblock towards high-performance devices are nanoscale variations of the graphene device properties, impacting their macroscopic behaviour. Here we present and apply non-invasive optoelectronic nanoscopy to measure the optical and electronic properties of graphene devices locally. This is achieved by combining scanning near-field infrared nanoscopy with electrical read-out, allowing infrared photocurrent mapping at length scales of tens of nanometres. Using this technique, we study the impact of edges and grain boundaries on the spatial carrier density profiles and local thermoelectric properties. Moreover, we show that the technique can readily be applied to encapsulated graphene devices. We observe charge build-up near the edges and demonstrate a solution to this issue.

## Abstract (web summary)

Graphene grain boundaries and charge inhomogeneities limit its electronic properties. Here the authors combine scanning near-field optical microscopy with electrical read-out to image these defects at the nanoscale under an encapsulation layer, and show that charges build up along the edges of the flake.

## Graphical abstract

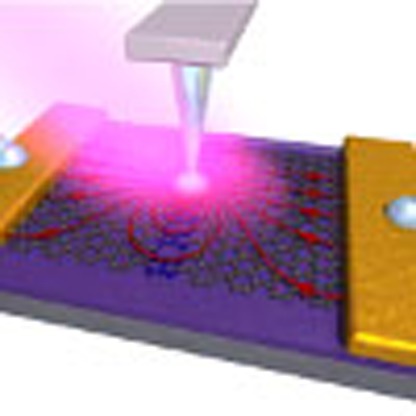

As large scale integration and wafer scale device processing capabilities of graphene have become available^1^,^2^,^3^,^4^,^5^,^6^,^7^,^8^, technological implementations of electronic and optoelectronic graphene devices are within reach^9^,^10^,^11^. At the same time, to achieve high device performance, any imperfections at the nanometer or even atomic scale need to be minimized or even eliminated. For example, in large area graphene, grown by chemical vapour deposition (CVD), grain boundaries are the stitching regions between different monocrystalline parts of graphene and act as carrier scatterers, limiting the graphene mobility and uniformity^12^,^13^. In addition, even perfectly monocrystalline graphene is still highly sensitive to its environment, and on typical substrates charge–density inhomogeneities (charge puddles)^14^,^15^,^16^,^17^,^18^,^19^ and additional doping near contacts, defects and edges arise, which reduce the device performance as well. Therefore, it is important to efficiently probe the nanoscale optoelectronic properties of graphene devices and to understand their microscopic physical behaviour.

A major challenge is that many of the available characterization techniques are invasive^20^, need specifically designed device structures^13^,^21^,^22^, image only very small areas^14^,^15^,^21^,^23^,^24^,^25^, rely on high doping of the graphene,^26^ require unhindered electrical access of the probe to the graphene^14^,^15^,^21^,^23^,^24^ or lack the desired nanometer resolution^27^ and are expensive and difficult to implement. For the direct quality control of graphene devices, a method that can image electrical and optical properties of graphene devices, at nanoscale resolution, without any special preparation and without modifying the devices is required.

Here we demonstrate fully non-invasive room-temperature scanning near-field photocurrent nanoscopy^28^,^29^,^30^,^31^,^32^,^33^,^34^,^35^,^36^,^37^,^38^ for the first time applied on graphene with infrared frequencies and use it to study the nanoscale optoelectronic properties of graphene devices that can later be used for real applications. This technique is based on electrical probing of the photoresponse due to strongly localized heating. We apply this technique to study the microscopic physics of grain boundaries and charge–density inhomogeneities. In the case of grain boundaries, we were able to identify the magnitude of their Seebeck coefficient, while for charge–density inhomogeneities, we show how they influence the global charge neutrality point of graphene devices. In addition, we study encapsulated graphene devices^39^,^40^, where the encapsulation would prevent many other scanning probe techniques from accessing local properties of graphene. In these devices, we find a charge build-up near the edges and show that using local metal gates instead of a global backgate effectively suppresses this type of edge doping. In general, this technique operates most effectively with mid-infrared light because it does not lead to photodoping^41^ and it is more stable in operation, compared with visible light.

## Results

### Measurement principle

**Figure 1:**
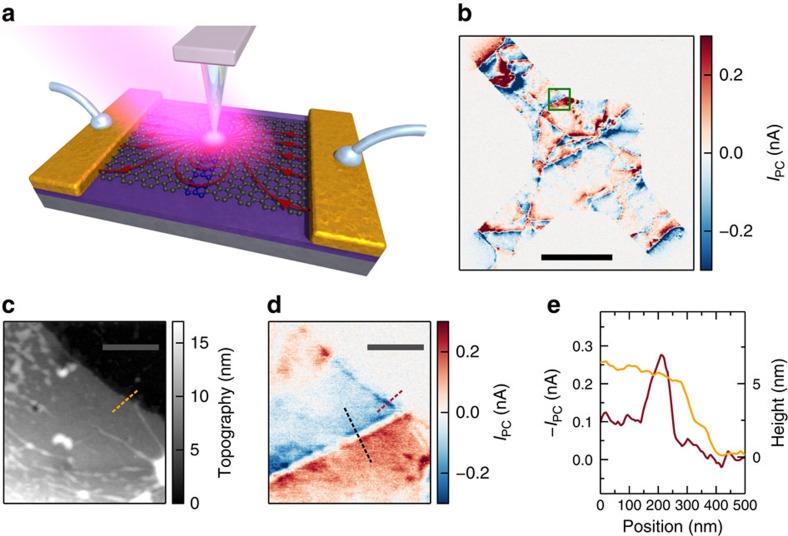
Near-field photocurrent working principle and photocurrent from grain boundaries. (**a**) Sketch of the scattering-type scanning near-field optical microscope setup. A mid-infrared laser illuminates the atomic force microscope tip, which generates a locally concentrated optical field, which is absorbed by the graphene generating a position dependent photocurrent. The blue region in the graphene lattice represents a grain boundary with a modified Seebeck coefficient. The arrows sketch the photocurrent flow pattern. For each position only the magnitude and direction of the current are measured. The sketch is not to scale. (**b**) *I*_PC_ map at at backgate voltage *V*_BG_=0 V of a single layer CVD graphene device (Supplementary Fig. 1) with three contacts: top left (drain), right (source) and bottom left (ground). Both grain boundaries and wrinkles show characteristic photocurrent patterns. (scale bar, 5 μm) The green box indicates the measurement region in **c**,**d**. (**c**) Topography of etched CVD graphene does not show grain boundary but only wrinkles and other inhomogeneities due to the transfer process. (scale bar, 500 nm) (**d**) *I*_PC_ at *V*_BG_=0 V clearly shows a grain boundary and the expected sign change around it. The black dashed line indicates the measurement positions in Fig. 2a,d. (scale bar, 500 nm) (**e**) Topography (orange) and *I*_PC_ (red) measured at the orange line in **c** and the red line in **d**, respectively.

The measurement principle is sketched in Fig. 1a. The setup is based on a scattering-type scanning near-field optical microscope (s-SNOM)^26^,^42^ augmented with electrical contact to the sample to measure currents *in situ*^28^,^29^,^30^,^31^,^32^,^33^,^34^,^35^,^36^,^37^,^38^. In contrast to conventional s-SNOM, we do not need to measure the outscattered light but rather directly measure current induced by the near-field as explained in the following. A 10.6-μm mid-infrared laser illuminates a metallized atomic force microscope probe, tapping at its mechanical resonance frequency. Part of the incoming light, polarized parallel to the shaft of the probe, excites a strong electric field at the tip apex due to an antenna effect^43^. The spatial extent of this near-field is on the order of 25nm, limited only by the tip radius and much smaller than the free space wavelength of the impinging light^43^.

The near and far fields impinging on the device induce charge flows in the device (by mechanisms discussed below), and drive currents into an external current amplifier via contacts on the device. We isolate the part of the current that is induced by near fields by demodulating the current at the second harmonic of the tip tapping frequency^43^. This demodulated current is denoted *I*_PC_ and referred to as near-field photocurrent and is obtained together with near-field optical and topography information. A typical map of *I*_PC_, obtained by scanning the tip over a CVD graphene device, is shown in Fig. 1b.

We can assess the spatial resolution of the photocurrent maps by comparing a region near the edge (Fig. 1d) with a topographic image from the same region (Fig. 1c). As can be seen, *I*_PC_ falls to zero for tip locations away from the graphene on a similar length scale as the topography, demonstrating the successful isolation of near-field contributions. In Fig. 1e, we quantify the resolution by observing the change in *I*_PC_ as the tip is moved over the edge of graphene. The full-width at half maximum of the photocurrent peak at this location is ∼100 nm, matching the rise distance in the topographic signal. This resolution is far below any limits relating to the 10.6 μm free space light wavelength.

### Photothermoelectric photocurrent generation mechanism

As to the physical mechanism of the photocurrent, we consider the photothermoelectric effect that has been shown to dominate the photoresponse of graphene^11^,^44^,^45^,^46^,^47^,^48^: the light (in this case, the tip-enhanced near-field) locally heats the graphene, and this heat acts via non-uniformities in Seebeck coefficient *S* to drive charge currents within the device and into the contacts (see Methods section). Therefore, we interpret the variations of *I*_PC_ in terms of microscopic variations in *S*. The Seebeck coefficient, which depends on material properties such as carrier density and mobility, is a measure of the electromotive force driven by a temperature difference in a material. A complete description of *I*_PC_ needs to take into account the carrier cooling length^45^,^46^ and overall sample geometry^49^. The carrier cooling length 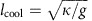, where *κ* the sheet thermal conductivity in plane and *g* the interfacial thermal conductivity out of plane to the heat sinking substrate, describes how far heat propagates through the charge carriers, before dissipating to the environment (see Supplementary Fig. 2)^46^. A quantitative model of the thermoelectric photocurrent mechanism can be found in the Methods section.

### Grain boundary characterization

We first discuss the application of this infrared near-field photocurrent technique to grain boundaries. They are not visible in the simultaneously acquired topography, and are responsible for some of the line-shaped features in the photocurrent map in Fig. 1b. Some of the other features stem from large scale inhomogeneities of the sample. The region within the green frame is shown with higher resolution in Fig. 1d, exhibiting a strong photocurrent signal that changes sign along a sharp boundary, yet the graphene is topographically flat in the vicinity of this boundary (Fig. 1c). We show now that this type of feature indicates a grain boundary.

**Figure 2:**
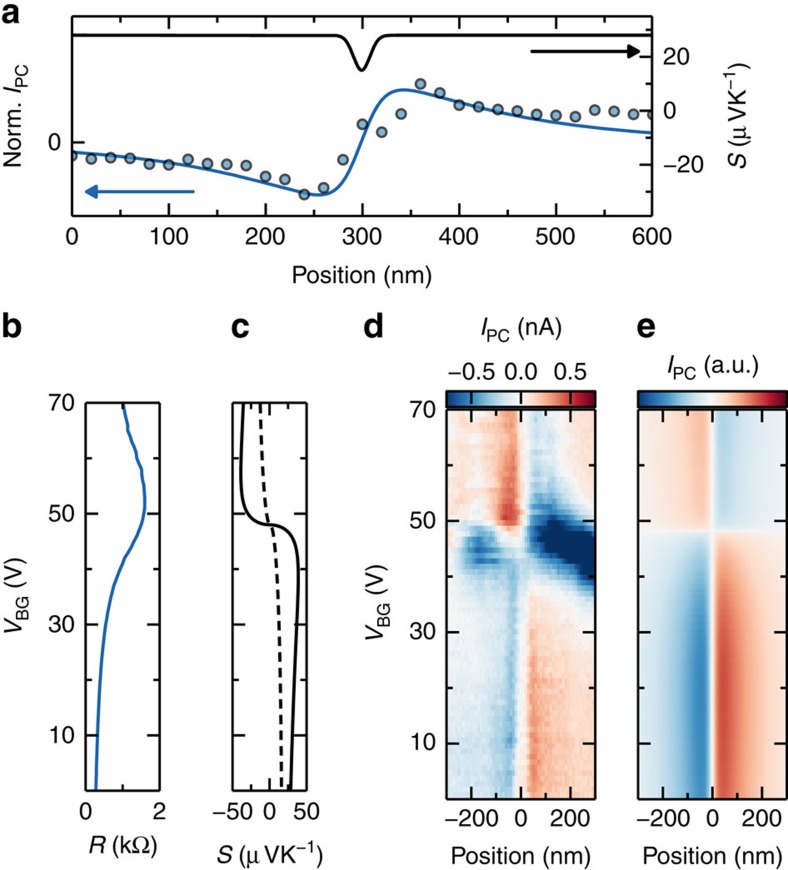
Photocurrent profile at a grain boundary and its gate voltage dependence. (**a**) Photocurrent profile, measured at the black dashed line in Fig. 1d, perpendicular to the grain boundary at *V*_BG_=0 V shows good agreement with the photothermoelectric model with *l*_cool_=140 nm. The modelled spatial Seebeck profile (with FWHM 20 nm) is shown in black. (**b**) Two-probe device resistance as a function of *V*_BG_. (**c**) Simulated Seebeck coefficient *S*_G_ for pristine graphene (solid line) and *S*_GB_ for polycrystalline graphene with an average grain size of 25 nm (dashed line; Supplementary Note 2). (**d**) Backgate dependent photocurrent profile perpendicular to the grain boundary shows that the grain boundary changes its sign at the charge neutrality point. (**e**) Simulated backgate dependent photocurrent profile based on the Seebeck profiles in **c** normalized to the simulated photocurrent maximum.

Figure 2a shows a line profile of *I*_PC_ across the boundary feature identified in Fig. 1d. This antisymmetric *I*_PC_ can be explained by a localized deviation in *S* at the boundary, that is, a line defect within an otherwise uniform thermoelectric medium (Supplementary Figs 3,4 and Supplementary Note 1). Indeed, grain boundaries behave as localized lines of strongly modified electronic properties, within otherwise uniform graphene^20^,^21^,^26^,^50^,^51^. We remark that the decay of the photocurrent away from the boundary extends over more than 100 nm, which is due to a larger hot carrier cooling. We find in this case *l*_cool_=140 nm.

To gain more insight in the Seebeck coefficient at the grain boundary, we tune the carrier density by a global gate (Fig. 2d). We observe that the antisymmetric spatial photocurrent profile changes sign as the backgate voltage *V*_BG_ passes the peak in resistance, that is, the global charge neutrality point *V*_D_. The Seebeck coefficient *S*_G_ of graphene itself changes sign at the charge neutrality point^44^,^45^,^52^,^53^ (Fig. 2c). Thus, after calibrating the sign to the known sign of the contact photocurrent, our data implies that the Seebeck coefficient of the grain boundary *S*_GB_ is always smaller in magnitude than *S*_G_, since *I*_PC_(*V*_BG_)∝*S*_G_(*V*_BG_)−*S*_GB_(*V*_BG_).

Using a polycrystalline graphene model, we compute the resistance due to grain boundaries using a Kubo transport formalism and real space simulations^54^. *S*_GB_ is the ratio of the first- and zero-order Onsager coefficients (Supplementary Note 2). Indeed, we find that *S*_GB_ is always smaller in magnitude and has a similar lineshape as *S*_G_ in the carrier density range measured (Fig. 2c). Figure 2e shows a simulation of the photocurrent for the calculated Seebeck coefficients, which is in agreement with the measurements (Supplementary Figs 5,6).

### Charge puddle characterization

**Figure 3:**
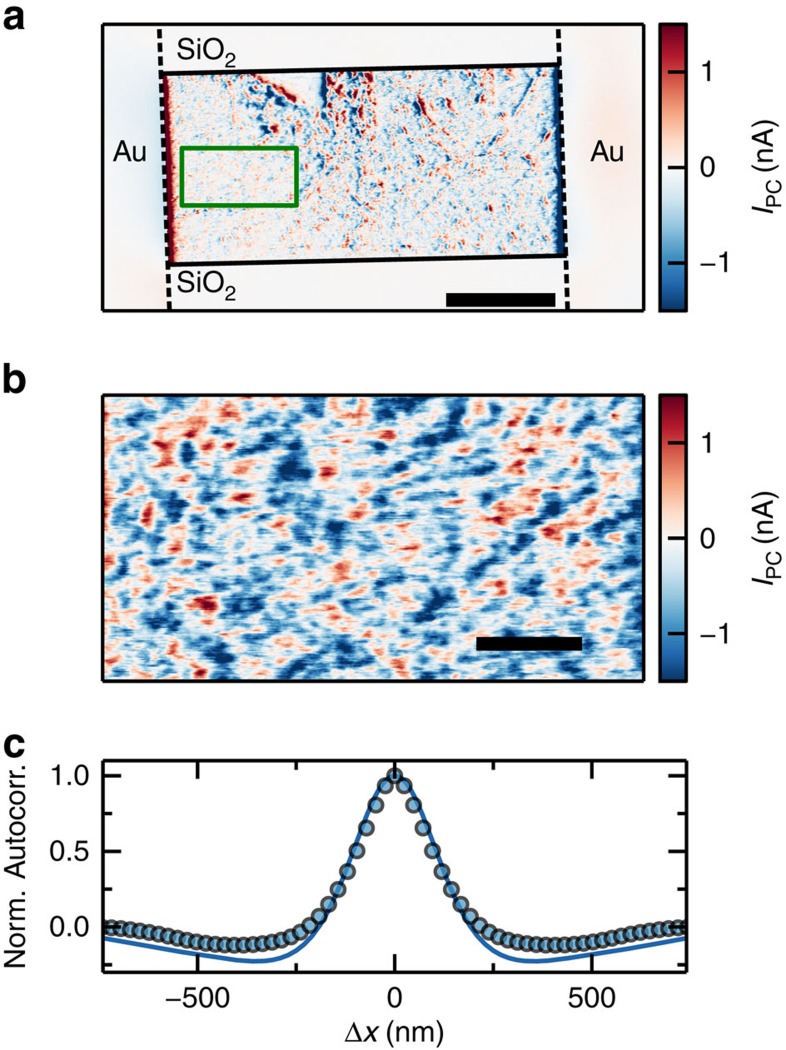
Photocurrent from charge puddles. (**a**) Near-field photocurrent map of an exfoliated graphene device on 300 nm SiO_2_ at *V*_BG_=20 V. The dashed lines indicate the position of the contacts and solid lines the graphene edges. The green box indicates the measurement region in **b** (scale bar, 5 μm). (**b**) Detailed photocurrent map at the charge neutrality point of the device (*V*_BG_=7 V) reveals the charge puddles and the high spatial resolution of the technique. (scale bar, 1 μm) (**c**) Autocorrelation of the photocurrent from charge puddles at *V*_D_ (data points) compared to photocurrent expected from a random charge puddle distribution and *l*_cool_=200 nm (blue curve). Autocorrelation is taken along the source drain current path.

We next examine near-field photocurrent in a typical two-probe exfoliated graphene device (Fig. 3). A strong photocurrent is obtained with the tip near the metal contacts, similar to previous near- and far-field measurements^34^,^47^,^55^. In addition, an apparently random pattern of photocurrent is present throughout the device, as in high-resolution far-field measurements^55^ but at a much finer scale.

The random photocurrent pattern between the contacts in Fig. 3a indicates random variations in Seebeck coefficient over short length scales (Supplementary Fig. 7). Random variations of the Seebeck coefficient are indeed expected since it depends on carrier density^52^, which in turn has fine-scaled inhomogeneities (charge puddles)^14^,^15^,^16^,^17^,^18^. The photocurrent variations can thus be used to gain insight in the charge puddle distribution. A more detailed view of the photocurrent due to charge puddles in Fig. 3b shows that the length scale that can be resolved is on the order of hundreds of nanometres.

Quantitatively, from the autocorrelation of the photocurrent in comparison with a photothermoelectric model taking into account the size of the charge puddles in Fig. 3c we extract *l*_cool_∼200 nm. The charge puddles are modelled to have a size of ∼20 nm, in accordance with measurements of graphene on silicon oxide (SiO_2_; refs 15, 16, 17, 18).

**Figure 4:**
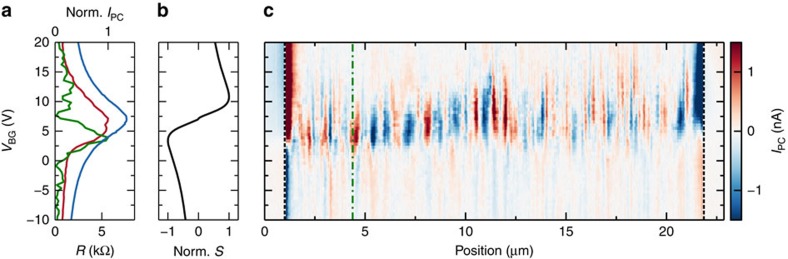
Dependence of photocurrent profiles on backgate voltage reveals doping inhomogeneities. (**a**) Backgate dependence of the resistance of the device measured simultaneously to the photocurrent in blue. The red curve shows the normalized root mean square of the photocurrent across the device. The green curve shows a single normalized photocurrent backgate trace, corresponding to the green dashed dotted line in **c**. (**b**) Backgate dependent Seebeck coefficient of graphene, calculated from the gate dependent resistance in **a** using the Mott formula^52^. (**c**) Backgate dependence of the photocurrent across the device. Graphene is between the black dashed lines, which indicate the edges of the metal contacts.

By changing the gate voltage we study the carrier density profile with high spatial resolution (Fig. 4) and highlight the possibility of spatially resolving the charge neutrality point for a large device. *I*_PC_ from charge puddles is largest around the charge neutrality point and varies with position. This is consistent with the very high sensitivity of the Seebeck coefficient to changes in carrier density, near-zero density (Fig. 4b). The magnitude of photocurrent from charge puddles depends on the difference of Seebeck coefficient between two adjacent charge puddles or in other words the strongest photocurrent from charge puddle appears at the position of highest Seebeck gradient. This allows us to map the local carrier density offset (charge inhomogeneity) throughout the device, as indicated by the extremum of photocurrent in a scan of photocurrent versus gate voltage (Fig. 4c). The photocurrent from adjacent charge puddles with a given charge carrier density offset does not change sign when sweeping through the charge neutrality point. This is because the difference in Seebeck coefficient between these puddles does not change sign.

We can thus resolve the local charge neutrality point at a given position of the device (green curve, Fig. 4a), which can be different from the global charge neutrality point *V*_D_, the backgate voltage *V*_BG_ at which the resistance is maximum (blue curve, Fig. 4a). We show that the global charge neutrality point (blue curve, Fig. 4a) is determined by an average of the gate voltages at which the local charge neutrality points appear (red curve, Fig. 4a). Spatially resolved puddle photocurrent can be much narrower (green curve, Fig. 4a) than the average of all possible current paths (red curve, Fig. 4a) This indicates that the graphene locally has less inhomogeneity. Thus the technique gives insight not only in the global but also in the local behaviour of the device.

### Characterization of encapsulated devices

**Figure 5:**
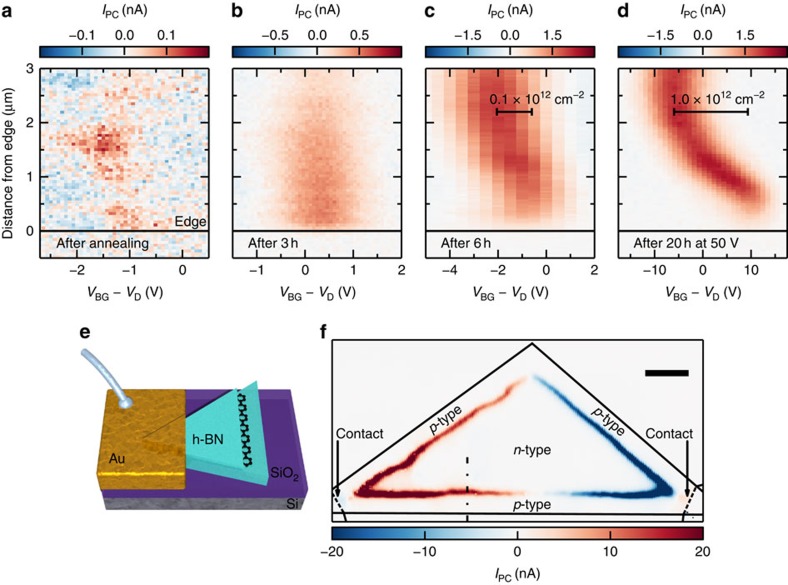
Near-field photocurrent maps revealing edge doping in encapsulated graphene. (**a**) Spatial photocurrent profile versus backgate voltage *V*_BG_ (minus voltage of the resistance maximum *V*_D_) near the edge of encapsulated graphene. These data are taken directly after annealing the device. (*V*_D_=−0.3 V). (**b**) The same scan on the same device after three hours in air, (*V*_D_=−0.5 V) and **c**, after annealing and applying *V*_BG_ up to 3 V (*V*_D_=3 V). (**d**) The same scan after approximately 20 h in air and after applying *V*_BG_ up to 50 V (*V*_D_=30 V). (**e**) Sketch of the device, a stack of h-BN(46 nm)/Graphene/h-BN(7 nm) on a Si/SiO_2_(300 nm) wafer used as global backgate. In this sketch we only show one of the two contacts used for electrical measurements. (**f**) Photocurrent close to the resistance maximum at *V*_BG_=−28 V shows a triangular photocurrent pattern, due to edge p-doping. The dashed dotted line indicates where the measurements in **a**–**d** were taken. (scale bar, 2 μm).

Finally, we apply this technique to a graphene device encapsulated between two layers of hexagonal boron nitride (h-BN), using the polymer-free van der Waals assembly technique^39^,^40^ as sketched in Fig. 5e. This device lies on top of an oxidized silicon wafer, used as a backgate. The stack is etched into a triangle and electrically side-contacted by two metal contacts^39^. Recent studies in vacuum and low temperatures^56^,^57^ have shown that the edges affect where current flows in the device, in particular near charge neutrality. In the following we study the build-up of edge doping in ambient conditions and provide a solution to this.

While monitoring the photocurrent of such encapsulated devices, we observe indications of strong carrier density variations near the edges over micrometre scales. These variations are influenced by lighting conditions (Supplementary Fig. 8), gate voltages, and temperature, and evolve over timescales ranging from minutes to weeks. As an example, Fig. 5a–d shows a progression of photocurrent maps, taken after annealing the device at 200 °C for 30 min in ambient conditions to temporarily remove charge density variations near the edges. Initially in Fig. 5a we see very small photocurrents indicating a flat carrier density landscape. After some time (hours), in the dark with only gate voltages smaller than 3 V applied, a small doping gradient between the contacts builds up. This gradient leads to the stronger photocurrent shown in Fig. 5b. The local charge neutrality point, indicated by the maximum of photocurrent, is at the same position close to the edge of the device as further inside the bulk. After keeping the device for 3 h in ambient conditions we can see a change of the local charge neutrality point at the edge of the graphene compared to the bulk in Fig. 5c. The edge is slightly more p-type compared to the bulk. Finally, we apply high gate voltages, of in this case 50 V for ∼20 h, to increase the edge doping. A strong p-doping at the edge and an n-doping in the bulk of the graphene is induced in Fig. 5d. This indicates that electric field accelerates the speed and increases this type of edge doping.

We exploit the observed edge doping to create a natural p–n junction along the edge of the device. For this we apply a backgate voltage at which the edge of the graphene is p-type and the bulk n-type. This is similar to the situation in Fig. 5d at *V*_BG_=*V*_D_. We observe photocurrent at the junction in Fig. 5f around the whole device, indicating that the edge doping is uniform around the graphene. The photocurrent decays gradually towards the midline between the electrodes as a result of how the triangular geometry modifies the ability of the contacts to capture photocurrents^49^. The sign change in the middle of the device is because in the current direction the junction changes from a p–n junction to an n–p junction (Supplementary Fig. 9 and Supplementary Note 2). We are able to temporarily reset the edge doping by annealing the device on a hotplate at 200 °C for 30 min as we show in Fig. 5a.

### Local gates prevent charge build-up at the device edges

**Figure 6:**
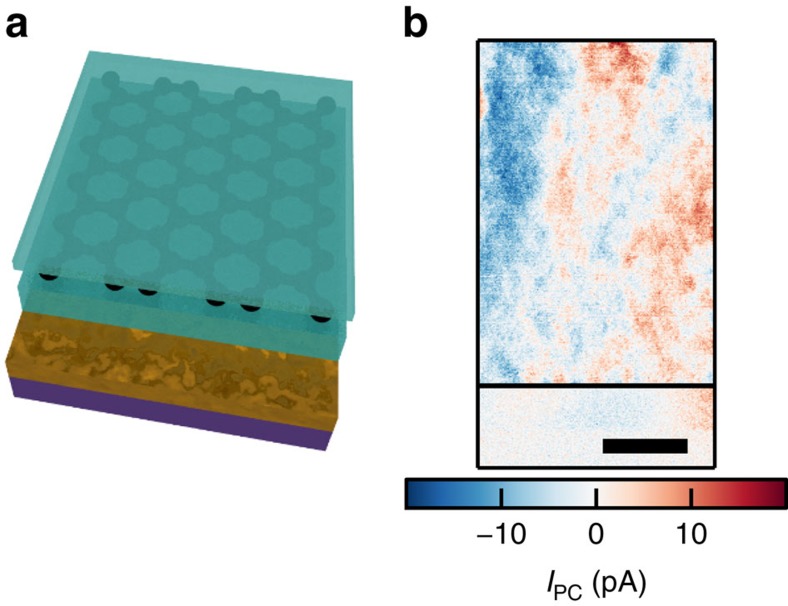
Edge doping is efficiently suppressed using devices with local metal gates. (**a**) Sketch of the device with local metal gate with the two h-BN layers in light blue and the local metal gate in gold. The layers are from bottom to top SiO_2_(300 nm)/AuPd(15 nm)/h-BN(42 nm)/Graphene/h-BN(13 nm). (**b**) Photocurrent from charge puddles in encapsulated graphene on a metal gate close to the charge neutrality point. The electrical contacts are on the left and right outside of this figure. (scale bar, 2 μm).

While we have not been able to precisely identify the origin of the edge doping, we suspect that water molecules are able to penetrate between the boron nitride and the SiO_2_ due to the surface of the h-BN being not completely conformal with the underlying SiO_2_ substrate. This water molecule penetration then leads to trapped charges is responsible for the observed edge doping. We also present here a technique to completely eliminate the edge doping. We place encapsulated graphene on top of a local conductive gate, such as a 15 nm AuPd alloy sketched in Fig. 6a. As the photocurrent measurement in Fig. 6b shows, we find that edge doping is efficiently suppressed even after extended periods of time at ambient conditions and high gate voltages. Furthermore, such devices efficiently suppress the photodoping effect observed for devices where the h-BN is in contact with SiO_2_ (ref. 41; Supplementary Fig. 10 and Supplementary Note 1).

In the device with a local metal gate used to suppress both edge- and photodoping, we find small features due to charge puddles on top of a slowly varying background photocurrent, due to large-scale carrier density inhomogeneities. The size of the features due to charge puddles determined by autocorrelation is ∼800 nm. The long length scale of those features is either due to the longer cooling length of the encapsulated graphene compared to the graphene on SiO_2_ or due to larger charge puddle size in the encapsulated devices. Further work is required to clearly distinguish these effects.

## Discussion

To conclude, we have demonstrated that scanning near-field photocurrent nanoscopy is a versatile technique to characterize the electronic and optoelectronic and even previously inaccessible properties of relevant graphene devices. This technique is highly promising for spatially resolved quality control of regular graphene devices without the need for special device structures and can therefore be readily applied.

## Methods

### Photocurrent model

Photocurrent *I*_PC_ in graphene as generated by the photothermoelectric effect and is described as:^45^,^46^,^47^


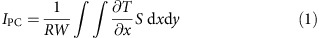


where *R* is the total resistance including graphene, contacts and circuitry, *W* the device width and *x* the current flow direction. This is valid for rectangular graphene devices and special care needs to be taken for arbitrary shapes, such as in Fig. 5 (ref. 49). For the temperature profile *T*(*x*) we consider that the heat spreads in two dimensions with heat sinking to lattice and substrate, producing a *T*(*x*) profile described by a modified Bessel function of the second kind, with a finite tip size correction (Supplementary Note 2). A 25-nm finite tip-size correction was used for all simulations.

### Measurement details

The s-SNOM used was a NeaSNOM from Neaspec GmbH, equipped with a CO_2_ laser operated at 10.6 μm, away from the phonon resonance of SiO_2_, which can lead to strong substrate contributions to the photocurrent^48^. The laser power used was ∼20 mW. The probes were commercially available metallized atomic force microscopy probes with an apex radius of approximately 25 nm. The tip height was modulated at a frequency of approximately 250 kHz with a 60–80 nm amplitude. A Femto DLPCA-200 current pre-amplifier was used. The probe tip was electrically grounded. Because of the different device geometries and the fact that all the measurements were taken at different times and slightly different device conditions the absolute values of the photocurrents are not comparable.

### Device fabrication

The CVD graphene, grown on copper, was transferred onto a self-assembled monolayer^58^ on 285 nm of SiO_2_ to stabilize the charge neutrality point. The contacts were defined using optical lithography with Ti (5 nm)/Pd (35 nm). The graphene was transferred onto deposited contacts.

The exfoliated graphene device was fabricated on a Si/SiO_2_(300 nm) wafer, used as backgate. The Cr(0.8 nm)/Au(80 nm) contacts were defined using electron beam lithography.

The Si/SiO_2_(300 nm)/h-BN(46 nm)/Graphene/h-BN(7 nm) and the Si/SiO_2_(300 nm)/AuPd(15 nm)/h-BN(42 nm)/Graphene/h-BN(13 nm) stacks, were fabricated using the polymer-free van der Waals assembling technique^39^.

## Additional information

**How to cite this article:** Woessner, A. *et al.* Near-field photocurrent nanoscopy on bare and encapsulated graphene. *Nat. Commun.* 7:10783 doi: 10.1038/ncomms10783 (2016).

## Supplementary Material

Supplementary Information Supplementary Figures 1-10, Supplementary Notes 1-2 and Supplementary References
