# Supplementary material for: Near-field photocurrent nanoscopy on bare and encapsulated graphene
Source: Nat Commun. 2016 Feb 26;7:10783. doi: 10.1038/ncomms10783 (PMC4773437; doi:10.1038/ncomms10783)
Supplement: Supplementary Information — Supplementary Figures 1-10, Supplementary Notes 1-2 and Supplementary References [file ncomms10783-s1.pdf]

SUPPLEMENTARY FIGURES

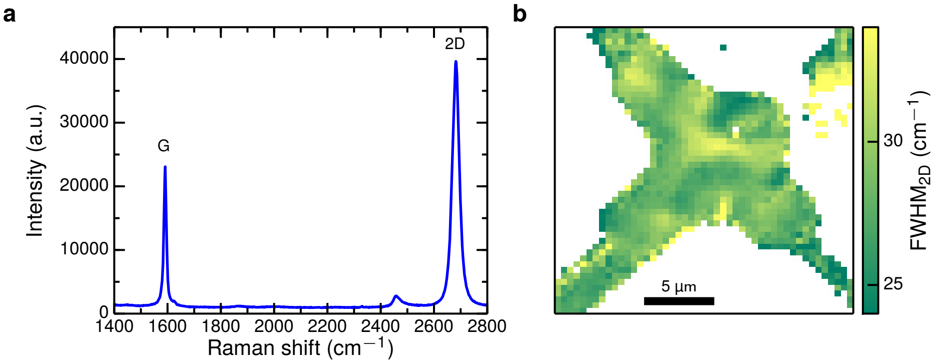

Supplementary Figure 1 | Raman spectrum and map of the CVD device. **a**, Raman spectrum of the graphene shows the characteristic G and 2D peak of single layer graphene. **b**, Map of the full width at half maximum of the 2D peak at the different positions of the device shown in Fig. 1b in the main text. This map clearly shows that the device is fully composed of single layer graphene. In the white region no graphene is present.

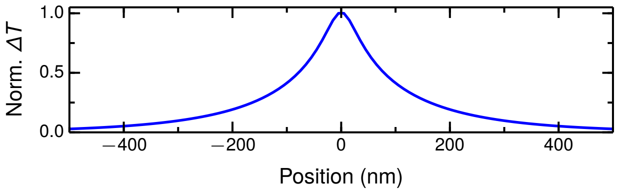

Supplementary Figure 2 | Steady state temperature profile created in infinite graphene with a thermal length of 200 nm and a finite tip size correction of 25 nm.

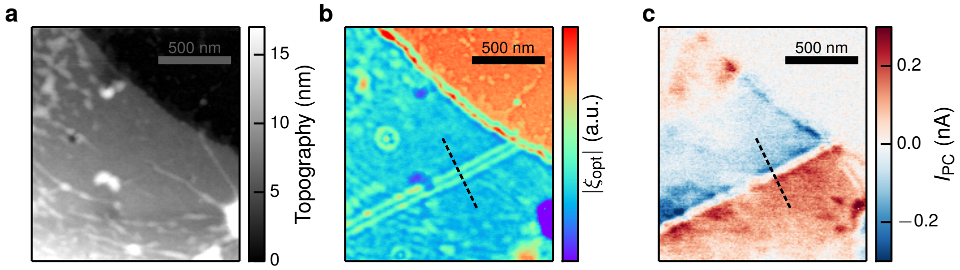

Supplementary Figure 3 | Comparison between topography, near-field optical scattering and near-field photocurrent. **a**, Topography of etched CVD graphene does not show grain boundaries but only wrinkles and other inhomogeneities due to the transfer process. **b**, Near-field optical scattering shows the characteristic plasmonic double fringes around a grain boundary in CVD graphene at $V_{BG} = 0$ V because the carrier density is $n_s = 3.7 \times 10^{12}$ cm^{-2} and plasmons are supported.[1,2] **c**, Near-field photocurrent clearly shows the grain boundary and a sign change around it. The dashed line in **b**,**c** indicates where the backgate dependent measurements of the near-field optical scattering and near-field photocurrent in Figs. 2c and d respectively in the main text where taken. All measurements of the near-field optical scattering ξ_{opt} presented here were obtained from the third harmonic interferometric pseudo-heterodyne signal,[3] measured with a cryogenic HgCdTe detector. For simplicity Supplementary Figure 3b only shows $|\xi_{opt}|$.

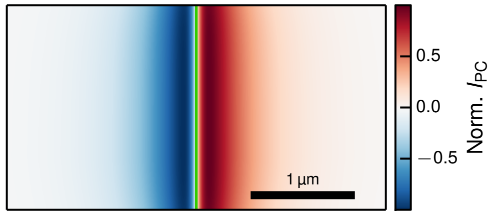

Supplementary Figure 4 | Simulated near-field photocurrent map from a grain boundary. A map of the simulated near-field photocurrent for each position of the graphene device. The contacts are on the left and right outside of the region shown in this figure. The cooling length used was 140 nm and the tip size 25 nm.

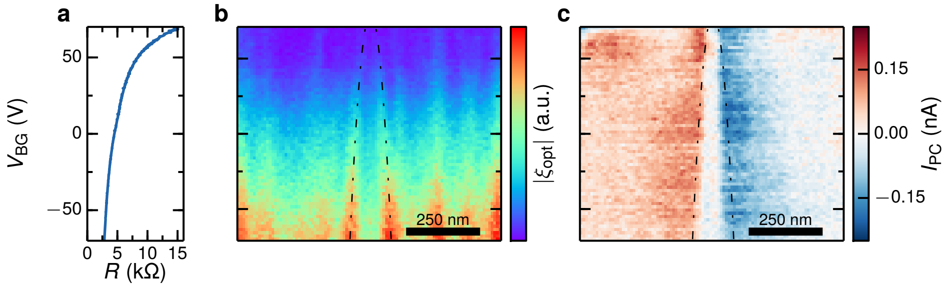

Supplementary Figure 5 | Gate dependent measurement of a grain boundary. a, Backgate dependence of the resistance of the device measured simultaneously to the near-field optical scattering and near-field photocurrent. **b**, Near-field optical scattering shows the plasmon reflections due to the grain boundary and their dependence on the carrier density of the graphene. **c**, Near-field photocurrent shows no sign change even for highly doped graphene. The dashed dotted curves in **b** and **c** show the theoretical fringe spacing for a phase shift due to the reflection of $-3/4\pi$.[1]

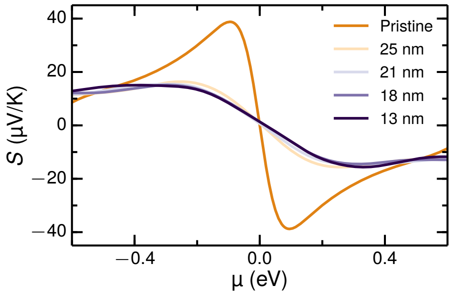

Supplementary Figure 6 | Simulated Seebeck coefficient for different polycrystalline samples ($T = 300$ K), for grain sizes (13, 18, 21 and 25 nm).

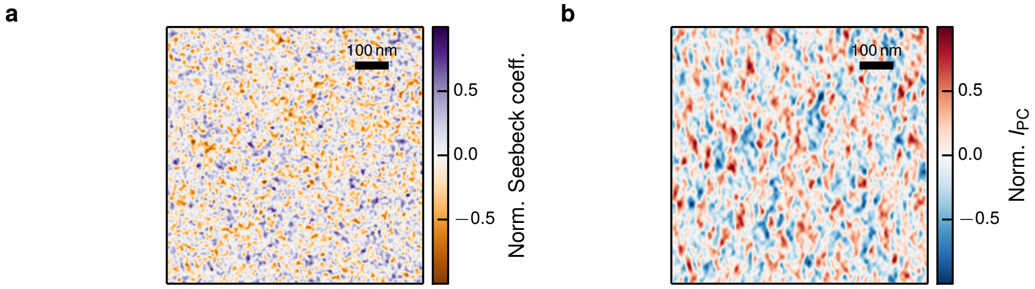

Supplementary Figure 7 | Simulation of a near-field phtocurrent map induced by a random charge puddle distribution. **a**, Random distribution of Seebeck coefficients due to a random distribution of charge puddles with an average size of $\sim$ 20 nm. **b**, Near-field photocurrent generated by the charge puddles in **a** with a cooling length of the charge carriers in the graphene of 200 nm and a tip size of 25 nm.

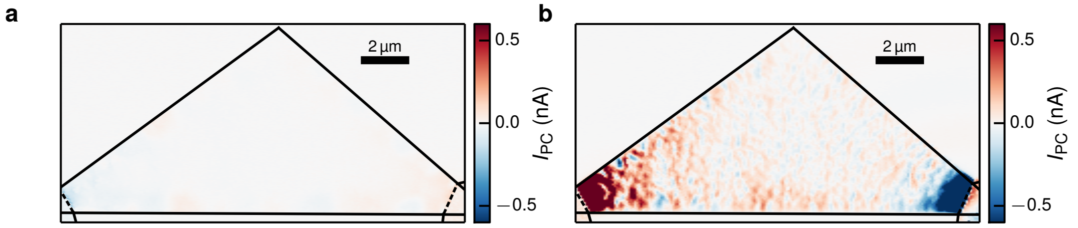

Supplementary Figure 8 | Encapsulated graphene device on oxidized silicon wafer. **a**, Near-field photocurrent of the graphene triangle device at -90 V backgate voltage in the dark. **b**, Photodoping showing up in the near-field photocurrent after illuminating the device for several minutes with white LED light at −90 V. The dashed lines in **a** and **b** indicate where the stack is underneath the gold and the solid lines the outer edge of the stack and gold respectively.

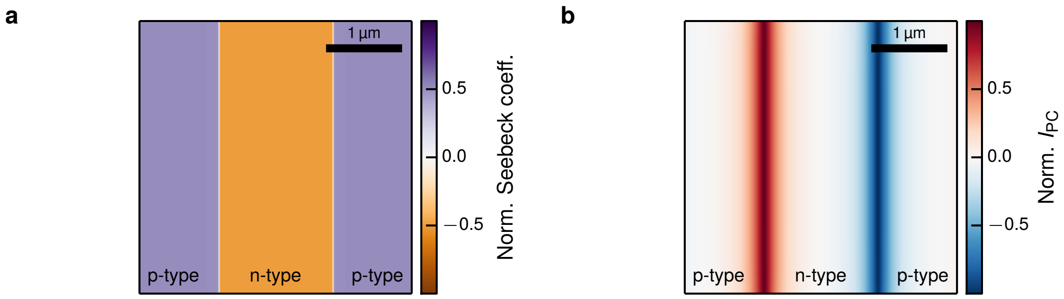

Supplementary Figure 9 | Simulation of a near-field photocurrent map from a pnp-junction. **a**, Spatial Seebeck coefficient profile for two p- and one n-type regions. The doping in the two regimes is assumed to be of the same magnitude and opposite sign. **b**, Near-field photocurrent generated by the pn- and np-junction with a cooling length of 200 nm and a tip size of 25 nm.

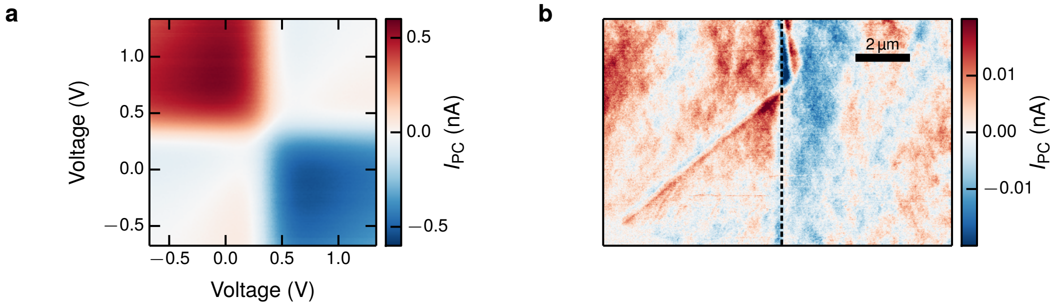

Supplementary Figure 10 | Encapsulated graphene with independently tunable gates. **a**, 6-fold pattern showing the thermoelectric origin of the near-field photocurrent at the junction between the two gates.[4] **b**, Near-field photocurrent from a local gated encapsulated graphene device with both sides of the local gate being tuned to a voltage of $V_{BG} = 0.4$ V close to the charge neutrality point. The layers are AuPd(15 nm)/h-BN(42 nm)/Gr/h-BN(13 nm). The dashed line indicates the position of the 50 nm gap between the two local gates.

Supplementary Note 1. MEASUREMENTS

In this section we show measurements that further support the proof that the antisymmetric near-field photocurrent pattern of Fig. 1 of the main text indeed stems from a grain boundary.

Raman spectra, topography, near-field optical scattering, and near-field photocurrent near a grain boundary

We show a map of the full width at half maximum of the 2D peak of the graphene Raman spectrum to confirm that the graphene is single layer everywhere on the device.[5] It is clearly visible that besides large scale inhomogeneities no fine features, as visible in the near-field photocurrent, due to grain boundaries are observed in the Raman map.

In Fig. 1c and d of the main text, which are reprinted here as Supplementary Figure 3a and c, we show the topography and the near-field photocurrent at a grain boundary for a backgate voltage of 0 V. To provide further evidence that the photocurrent stems from a grain boundary, we also show the simultaneously acquired near-field optical scattering data in Supplementary Figure 3b. The near-field optical scattering data is acquired using a conventional scattering-type scanning near-field optical microscope (s-SNOM).[6] It is a measure of the light that is scattered out of the graphene that interacts with the tip and is consequently detected in the far-field. The near-field optical scattering data show the typical double fringe due to plasmon reflections at the grain boundary.[1,2]

Even though conventional mid-infrared s-SNOM also offers the ability to detect grain boundaries in the near-field optical scattering,[1] there are some distinct advantages to using the near-field photocurrent. In order to observe plasmons due to grain boundaries, the graphene needs to be highly doped, as plasmons in graphene only propagate at at elevated carrier densities.[3,7,8] For small carrier densities plasmons are heavily damped and no double fringe is visible, as observed by a decreasing visibility of the double fringe near-field optical scattering close to the charge neutrality point of the graphene (see Supplementary Figure 5b).

In contrast, the near-field photocurrent shows a clear signature of the grain boundary even for small carrier densities (Supplementary Figure 5c), which enables us to extract more information, as discussed in the main text. This demonstrates further that near-field photocurrent is a useful tool to provide insight into the local properties of graphene. The near-field photocurrent technique also facilitates measurements compared to measuring the near-field optical scattering as no weak outscattered light needs to be collected and no interferometric measurement is required.[2,9]

When comparing the topography in Supplementary Figure 3a with the near-field optical scattering in Supplementary Figure 3b and the near-field photocurrent in Supplementary Figure 3c it becomes evident that the near-field optical scattering and the near-field photocurrent contain information that is not visible in the topography.

Gate dependence of a grain boundary

As explained in the main text and shown in Fig. 2c, the Seebeck coefficient at or very near the grain boundary is smaller in magnitude than the Seebeck coefficient of the surrounding pristine graphene for all the carrier densities measured. To further support this statement, we show the near-field optical scattering and near-field photocurrent for an extended carrier density range in Supplementary Figure 5. These data show that there is no additional sign change for higher carrier densities indicating that the Seebeck coefficient at the grain boundary S_{GB} is smaller in magnitude than the Seebeck coefficient of pristine graphene S_{G} for the measurable range of carrier densities. The measurements where done on the same device and grain boundary as the ones shown in the main text. We note that in the case of Supplementary Figure 5c there is no sign change as a function of gate voltage visible as the charge neutrality point of the device was not reached due to high intrinsic doping.

When comparing the position of the photocurrent extrema in Supplementary Figure 5c with the expected plasmonic fringe spacing as indicated by the dashed dotted curve it becomes evident indeed also the position of the photocurrent extrema is changing with carrier density. This could be due to an increased absorption due to the excitation of plasmons in the graphene and is subject of further study.

Photodoping and puddles of encapsulated graphene

It is known that graphene on h-BN on top of an oxidized silicon wafer shows photodoping when it is illuminated with visible light.[10] This effect is clearly visible in our near-field photocurrent measurements after illumination with visible light. In Supplementary Figure 8a we see almost no near-field photocurrent at a backgate voltage of −90 V. In Supplementary Figure 8b we show the near-field photocurrent pattern after the sample was illuminated with a white

LED light source for several minutes at a backgate voltage of −90 V. Clearly, near-field photocurrent is visible all the way throughout the device. We attribute this to the screening of the backgate by photoexcited defects in the h-BN or at the h-BN/SiO2 interface, which can effectively neutralize the graphene.[10] Thus we see charge puddles which show a very similar near-field photocurrent pattern compared to the near-field photocurrent from charge puddles observed at the charge neutrality point of exfoliated graphene on SiO_2 shown in Fig. 3 and Fig. 4 of the main text. In the case of the encapsulated device the charge puddles are induced by the photoexcited charged defects in the h-BN. In order to reset the photo doping and be immune to it we use positive gate voltages.[10]

Encapsulated graphene with a local gate

We also studied near-field photocurrent from an encapsulated device which was put onto conductive PdAu alloy gates with a 50 nm gap in between them in order to individually tune the carrier density in the graphene above the two gates. In Supplementary Figure 10a we show a six-fold pattern typical for a photothermoelectric effect[4] in graphene measured at the junction between the two gates, which is indicated by the dashed line in Supplementary Figure 10.

In Supplementary Figure 10b the current in the device is shown for both sides being tuned to a gate voltage of 0.4 V, close to the charge neutrality point. The magnitude of the near-field photocurrent is two orders of magnitude smaller than the magnitude of near-field photocurrent from charge puddles in graphene on SiO_2. This indicates that the charge density inhomogeneity of the charge puddles in encapsulated graphene on a local gate is much lower than for graphene on SiO_2. Furthermore the length scale of the charge puddles indicates that the charge puddle size is much larger than for the case of graphene on SiO_2[11,12] or that the cooling length in encapsulated graphene devices is much longer than for bare graphene, as expected due to the increased carrier mobility in encapsulated graphene.

We remark the we do not see any photodoping for the encapsulated graphene on top of a conductive gate structure, even after extended periods of exposure of many minutes to the same white light LED as was used to induce photodoping in Supplementary Figure 8b. This can be explained by the extraction of the photoexcited charged defects by the conductive gate, which is in direct contact with h-BN.

Supplementary Note 2. MODELLING

In this section we give a more detailed overview of the photothermoelectric photocurrent model used to describe the measurements and models in the main text.

Photothermoelectric model

Near-field photocurrent I_{PC} in graphene is governed by the photothermoelectric effect, which can be calculated by:[4,13,14]

$$I_{\mathrm{PC}}(x,y) = \frac{1}{RW}\int \frac{\partial T(x,y)}{\partial x} S(x,y)\, dx dy \tag{1}$$

where R is the total resistance, consisting of graphene, contact and circuit resistance, W the width of the device, $\partial T/\partial x$ is the gradient of the temperature T in current flow direction x and S is the Seebeck coefficient. In order to simulate the near-field photocurrent in arbitrary geometries special care has to be taken.[15]

For calculating I_{PC} at each position one has to do a convolution of the spatial Seebeck coefficient profile with the temperature gradient. This is numerically very expensive and the simulations can take a long time. In order to more efficiently simulate a near-field photocurrent map for an arbitrary spatial Seebeck profile we use the convolution theorem. This allows us to just multiply the Fourier transform of the spatial Seebeck coefficient and temperature gradient, multiply them and inverse Fourier transform them. This is computationally much faster and the simulation time is greatly reduced. This method was employed for all simulations shown in the main text as well as the Supplement.

Heat spreading in 2D

In order to describe the near-field photocurrent in graphene with a model it is of great importance to correctly describe the heat profile within the graphene as the heat profile in combination with the Seebeck profile lead to the near-field photocurrent pattern.

Graphene on top of a substrate is a two dimensional material with the substrate acting as a heat sink, as for typical substrate materials such as SiO_2 the thermal conductivity is much larger than for air. The two dimensional heat equation in steady state with an additional term for heat sinking can be written as:

$$0 = \kappa \nabla^2 T + P - g(T - T_\mathrm{s}), \tag{2}$$

where T is the electron temperature, T_s is the constant heat sink temperature, κ the thermal conductivity in plane, g the thermal conductivity out of plane to the heat sinking substrate and P the power density of the heat source.

We apply this equation by assuming that the electron heat cools to the lattice, but then that the lattice can pass on any heat much more easily to substrate. In that way the temperature of the lattice does not change significantly. This is convenient because the lattice itself has a different thermal length which would complicate matters.

If we take the two-dimensional case and a point source for P,

$$P = P_\mathrm{total}\delta(x)\delta(y), \tag{3}$$

then the solution of the two dimensional heat eq. 2 turns out to be:

$$T_\mathrm{spot} = T_0 K_0 \left(\frac{|r|}{l_\mathrm{cool}} \right) \tag{4}$$

where K_0 is the modified Bessel function of the second kind, $r = \sqrt{x^2 + y^2}$, the cooling length $l_\mathrm{cool} = \sqrt{\kappa/g}$ and the maximum temperature rise $T_0 = P_\mathrm{total}/(2\pi\kappa)$.

If we also include l_tip to approximate the effect of the finite tip size we end up with a heat spot of the following form:

$$T_\mathrm{spot}\left(x' - x, y' - y\right) = T_0 K_0 \left(\sqrt{\frac{(x'-x)^2 + (y'-y)^2 + l_\mathrm{tip}^2}{l_\mathrm{cool}^2}} \right) \tag{5}$$

A typical temperature profile as calculated by eq. 5 with a finite tip size approximation $l_\mathrm{tip} = 25$ nm, the actual radius of the tip, and a cooling length of $l_\mathrm{cool} = 200$ nm is presented in Supplementary Figure 2.

Grain boundary model

Grain boundaries can be modelled as having a finite width with a Gaussian profile[1] and their Seebeck coefficient is smaller in magnitude than the one of the surrounding pristine graphene, in accordance with the results of the main text. The near-field photocurrent profile is then calculated by performing a two dimensional convolution between the temperature profile defined by eq. (5) and the Seebeck profile. The results of this convolution are shown in Supplementary Figure 4.

Seebeck coefficient of polycrystalline graphene

We use an order-N Kubo-Greenwood wavepacket approach to calculate the conductivity of polycrystalline samples with different average grain sizes,[16] and we use square samples to convert this conductivity to conductance (G). Additionally, we calculate the conductance of pristine graphene using a Landauer approach. The Seebeck coefficient is calculated as the ratio of the first- and zero-order Onsager coefficients,

$$S(\mu, T) = -\frac{1}{|e|T} \frac{\int_{-\infty}^{\infty} (E - \mu) G(E) \left(-\frac{\partial f}{\partial E} \right) dE}{\int_{-\infty}^{\infty} G(E) \left(-\frac{\partial f}{\partial E} \right) dE}, \tag{6}$$

where f is the Fermi-Dirac distribution, μ the chemical potential and we set the temperature T to 300 K.

For polycrystalline samples with different average grain size (13, 18, 21 and 25 nm) we find that the Seebeck coefficient is significantly reduced compared to the clean case due to scattering at the grain boundaries (Figure 6). Furthermore, the Seebeck coefficient is independent of grain size, resulting from the linear scaling of charge transport in polycrystalline graphene[16,17]. The impact of the grain boundaries is reduced for larger chemical potentials (> 0.3 eV), where the Seebeck coefficient for polycrystalline and pristine graphene is similar.

The simulation of the backgate dependent near-field photocurrent in Fig. 2e of the main text was performed by using a Gaussian by using a Gaussian spatial distribution of the Seebeck coefficient with a full width at half maximum of 20 nm. The difference in Seebeck coefficient between S_{G} of the pristine graphene and S_{GB} at the grain boundary was calculated for each measured backgate voltage. For S_{GB}, the simulation corresponding to 25 nm polycrystalline graphene was used, but the results are essentially the same for different grain sizes. The spatial near-field photocurrent profile for each backgate voltage was calculated according to the procedure given in Supplementary Note 2 and then normalized by the resistance R for each gate voltage according to eq. (1).

Charge puddle model

In order to model near-field photocurrent from charge puddles, we first need to find an accurate model of the charge puddle distribution. For this we use a random spatial Seebeck profile generated by a spatial profile of white noise and smoothing the noise to create charge puddles with an average approximate size of 20 nm. This size was extracted from previous experimental studies of charge puddles on SiO_2.[11,12,18,19]

We calculate the near-field photocurrent map from the charge puddles. To this end, we again convolve the spatial Seebeck profile with the spatial heat gradient profile. A comparison between the spatial Seebeck profile and the near-field photocurrent map is shown in Supplementary Figure 7. Here the source and drain contacts are outside of the display on the left and right respectively. It is obvious that for positions with a high gradient in Seebeck coefficient the near-field photocurrent is strongest, as expected from eq. 1.

Model of a pnp-junction

Here, we model the near-field photocurrent map of a pnp-junction, where the p-type and n-type regions are extended to a size larger than the heat spot size. The transition length scale of the regions is smaller than the heat spot size. In this case the near-field photocurrent at the pn- and np-junction respectively has a single sign as observed for example in the triangular near-field photocurrent pattern due to edge doping in Fig. 5f in the main text. In the simulations shown in Supplementary Figure 9a and b the electrical contacts are on the left and right outside of the shown region.

SUPPLEMENTARY REFERENCES

[1] Fei, Z. et al. Electronic and plasmonic phenomena at graphene grain boundaries. *Nature Nanotech.* **8**, 821–825 (2013).
[2] Schnell, M., Carney, P. S. & Hillenbrand, R. Synthetic optical holography for rapid nanoimaging. *Nat. Commun.* **5**, 3499 (2014).
[3] Chen, J. et al. Optical nano-imaging of gate-tunable graphene plasmons. *Nature* **487**, 77–81 (2012).
[4] Gabor, N. M. et al. Hot carrier-assisted intrinsic photoresponse in graphene. *Science* **334**, 648–652 (2011).
[5] Ferrari, A. C. & Basko, D. M. Raman spectroscopy as a versatile tool for studying the properties of graphene. *Nature Nanotech.* **8**, 235–246 (2013).
[6] Keilmann, F. & Hillenbrand, R. Near-field microscopy by elastic light scattering from a tip. *Phil. Trans. R. Soc. A* **362**, 787–805 (2004).
[7] Fei, Z. et al. Gate-tuning of graphene plasmons revealed by infrared nano-imaging. *Nature* **487**, 82–85 (2012).
[8] Woessner, A. et al. Highly confined low-loss plasmons in graphene–boron nitride heterostructures. *Nature Mater.* **14**, 421–425 (2015).
[9] Ocelic, N., Huber, A. & Hillenbrand, R. Pseudoheterodyne detection for background-free near-field spectroscopy. *Appl. Phys. Lett.* **89**, 101124 (2006).
[10] Ju, L. et al. Photoinduced doping in heterostructures of graphene and boron nitride. *Nature Nanotech.* **9**, 348–352 (2014).
[11] Xue, J. et al. Scanning tunnelling microscopy and spectroscopy of ultra-flat graphene on hexagonal boron nitride. *Nature Mater.* **10**, 282–285 (2011).
[12] Decker, R. et al. Local electronic properties of graphene on a BN substrate via scanning tunneling microscopy. *Nano Lett.* **11**, 2291–2295 (2011).
[13] Song, J. C. W., Rudner, M. S., Marcus, C. M. & Levitov, L. S. Hot carrier transport and photocurrent response in graphene. *Nano Lett.* **11**, 4688–4692 (2011).

[14] Tielrooij, K. J. et al. Hot-carrier photocurrent effects at graphene–metal interfaces. *J. Phys. Condens. Matter* **27**, 164207 (2015).

[15] Song, J. C. W. & Levitov, L. S. Shockley-Ramo theorem and long-range photocurrent response in gapless materials. *Phys. Rev. B* **90**, 075415 (2014).

[16] Van Tuan, D. et al. Scaling properties of charge transport in polycrystalline graphene. *Nano Lett.* **13**, 1730–1735 (2013).

[17] Cummings, A. W. et al. Charge transport in polycrystalline graphene: challenges and opportunities. *Adv. Mater.* **26**, 5079–5094 (2014).

[18] Chen, J.-H., Jang, C., Xiao, S., Ishigami, M. & Fuhrer, M. S. Intrinsic and extrinsic performance limits of graphene devices on SiO2. *Nature Nanotech.* **3**, 206–209 (2008).

[19] Zhang, Y., Brar, V. W., Girit, C., Zettl, A. & Crommie, M. F. Origin of spatial charge inhomogeneity in graphene. *Nature Phys.* **5**, 722–726 (2009).
